# Identification of Micro Ribonucleic Acids and Their Targets in Response to *Plasmodiophora brassicae* Infection in *Brassica napus*

**DOI:** 10.3389/fpls.2021.734419

**Published:** 2021-10-28

**Authors:** Qian Li, Nadil Shah, Xueqing Zhou, Huiying Wang, Wenlin Yu, Jiajie Luo, Yajun Liu, Genze Li, Chao Liu, Chunyu Zhang, Peng Chen

**Affiliations:** ^1^College of Plant Science and Technology, Huazhong Agricultural University, Wuhan, China; ^2^Agricultural Technology Extension Station of Linxiang, Lincang, China; ^3^Agricultural Technology Extension Station of Lincang, Lincang, China; ^4^Industrial Crops Institute of Yunnan Academy of Agricultural Sciences, Kunming, China

**Keywords:** *Plasmodiophora brassicae*, clubroot resistance, miRNA, degradome, *Brassica napus*

## Abstract

Clubroot disease, which is caused by the soil-borne pathogen *Plasmodiophora brassicae* War (*P. brassicae*), is one of the oldest and most destructive diseases of *Brassica* and cruciferous crops in the world. Plant microRNAs [micro ribonucleic acids (miRNAs)] play important regulatory roles in several developmental processes. Although the role of plant miRNAs in plant-microbe interaction has been extensively studied, there are only few reports on the specific functions of miRNAs in response to *P. brassicae*. This study investigated the roles of miRNAs and their targets during *P. brassicae* infection in a pair of *Brassica napus* near-isogenic lines (NILs), namely clubroot-resistant line 409R and clubroot-susceptible line 409S. Small RNA sequencing (sRNA-seq) and degradome-seq were performed on root samples of 409R and 409S with or without *P. brassicae* inoculation. sRNA-seq identified a total of 48 conserved and 72 novel miRNAs, among which 18 had a significant differential expression in the root of 409R, while only one miRNA was differentially expressed in the root of 409S after *P. brassicae* inoculation. The degradome-seq analysis identified 938 miRNA target transcripts, which are transcription factors, enzymes, and proteins involved in multiple biological processes and most significantly enriched in the plant hormone signal transduction pathway. Between 409R and 409S, we found eight different degradation pathways in response to *P. brassicae* infection, such as those related to fatty acids. By combining published transcriptome data, we identified a total of six antagonistic miRNA-target pairs in 409R that are responsive to *P. brassicae* infection and involved in pathways associated with root development, hypersensitive cell death, and chloroplast metabolic synthesis. Our results reveal that *P. brassicae* infection leads to great changes in miRNA pool and target transcripts. More interestingly, these changes are different between 409R and 409S. Clarification of the crosstalk between miRNAs and their targets may shed new light on the possible mechanisms underlying the pathogen resistance against *P. brassicae*.

## Introduction

Rapeseed (*Brassica napus*) is an important oilseed crop in the temperate climate zone of the world, providing edible oil and raw materials for the production of bioenergy (Zajac et al., 2016). China is the second-largest producer of rapeseed, which is the fourth leading cash crop after rice, wheat, and maize (Hu et al., 2017). Rapeseed is also widely cultivated in the European Union, Canada, and other parts of Asia (Zajac et al., 2016).

Clubroot is a disease caused by the soil-borne pathogen *Plasmodiophora brassicae* War (*P. brassicae*) and one of the oldest and most destructive diseases of *Brassica* and cruciferous crops in the world (Dixon, 2014; Hirani et al., 2016). It spreads in more than 60 countries and causes more than 20% yield loss in highly infested fields (Diederichsen et al., 2009; Bhattacharya et al., 2014; Chai et al., 2014; Rahman et al., 2014; Wallenhammar et al., 2014). The pathogen can cause the formation of galls or clubs on the roots of susceptible hosts, which prevents water and nutrient uptake from the soil and finally results in stunting, wilting, and immature death (Dixon, 2009; Hwang et al., 2011). Besides, this obligate biotrophic pathogen can survive on soil for more than 20 years, resulting in very difficult control of the disease with chemicals or other mechanical methods (Kageyama and Asano, 2009). Therefore, the development of *P. brassicae*-resistant varieties is considered the most economical and effective approach to control the clubroot disease. To date, a number of clubroot resistant (CR) loci have been identified, such as *CRa, CRb, CRc, CRk* (Matsumoto et al., 1998; Piao et al., 2004; Sakamoto et al., 2008), *Crr1, Crr2, Crr3, Crr4* (Suwabe et al., 2003, 2006; Hirai et al., 2004), *CRd* (Pang et al., 2018), *PbBa3.1, PbBa3.2, PbBa3.3, PbBa1.1, PbBa8.1* (Chen et al., 2013), *Rcr1* (Chu et al., 2014), *Rcr4, Rcr8*, and *Rcr9* (Yu et al., 2017). Among these loci, *CRa* and *Crr1* are isolated from Chinese cabbage and encode toll-interleukin-1 receptor/nucleotide-binding site/leucine-rich-repeat (TNL/TIR-NBS-LRR) proteins (Hatakeyama et al., 2013, 2017). These resistance-related proteins (R proteins) are mostly intracellular receptors that interact with pathogen “effectors” to activate the effect or triggered immunity (ETI) of plants (Jones and Dangl, 2006). However, the specific mechanism for resistance to *P. brassicae* mediated by clubroot resistance (CR) genes remains unclear.

Micro ribonucleic acids (miRNAs) are a class of endogenous non-coding small RNAs usually with a length of 20–24 nucleotides (nt). miRNAs have been demonstrated to play important regulatory roles in several plant growth and development processes (Sunkar et al., 2012; Jin et al., 2013; Tang and Chu, 2017; Song et al., 2019). In plants, the miRNA-mediated regulation of gene expression occurs in three ways. First, miRNAs can directly target the messenger RNAs based on near-perfect sequence complementarity and lead to the cleavage of the targets (Song et al., 2004; Baumberger and Baulcombe, 2005; German et al., 2008; Carbonell et al., 2012; Fei et al., 2013). Second, miRNAs can also downregulate gene expression through translational repression that reduces protein level (Brodersen et al., 2008; Iwakawa and Tomari, 2013; Li et al., 2013; Reis et al., 2015). Third, besides the cleavage of target miRNAs and translational repression at the posttranscriptional level, miRNAs can also influence the level of transcripts through DNA methylation (Bao et al., 2004; Wu et al., 2010).

In the past few years, miRNAs have also been demonstrated to play crucial roles in mediating plant immune responses (Song et al., 2019; Kulshrestha et al., 2020). Generally, plants have two types of immune responses upon pathogen attack, which are known as the pathogen-associated molecular pattern (PAMP)-triggered immunity (PTI) and effector-triggered immunity (ETI) pathways (Jones and Dangl, 2006; Boller and Felix, 2009; Dangl, 2013; Peng et al., 2018). So far, at least 21 miRNA-target modules have been found to be involved in plant defense against pathogens through the regulation of PTI and ETI (Song et al., 2019). miR393 is the first miRNA identified in *Arabidopsis* for PTI induced by bacterial flagellin peptide, which negatively regulates auxin signaling by targeting the mRNAs of auxin receptors (Navarro et al., 2006). miR393 and miR166 are induced in the PTI of soybean roots upon infection by the fungus-like pathogen *Phytophthora sojae* (Wong et al., 2014). Hvu-miR398 is regulated by barley R genes *Mla* and *Rom1* and acts as a repressor of HvSOD1 in response to the barley powdery mildew fungus (Xu et al., 2014). miRNA393^*^ (derived from the lagging strand of pre-miR393), which is induced by avirulent *P. syringae pv*. Tomato DC3000 mediates the silencing of a Golgi-localized SNARE gene (*MEMB12)* and contributes to ETI in *Arabidopsis* (Zhang et al., 2011). However, R-protein-triggered ETI usually has a fitness cost for plant growth and, thus, is tightly controlled in the absence of pathogen attack and attenuated after defense (Tian et al., 2003; Deng et al., 2017; Greene and Dong, 2018; Wang et al., 2018; Liu et al., 2019; Cui et al., 2020). Recent studies have revealed that several miRNA families target the transcripts of R genes, which triggers the production of 21-nt phased siRNAs (phasiRNA), and prevent R-protein-triggered autoimmunity in the absence of pathogen infection (Zhai et al., 2011; Li et al., 2012; Shivaprasad et al., 2012; Liu et al., 2014; Gonzalez et al., 2015; Deng et al., 2018). Therefore, the miRNA-mediated regulation of R gene expression may be a conserved mechanism underlying pathogen-induced plant immunity (de Vries et al., 2015; Zhang et al., 2016).

Numerous studies have reported the functions of miRNAs in plant-microbe interactions. However, there have only been relatively few studies concerning the functions of miRNAs in response to *P. brassicae* infection in *B. napus*. Previous studies have reported miRNA expression profiles in *B. napus* or *Brassica rapa* under *P. brassicae* infection (Verma et al., 2014; Tang et al., 2015; Wei et al., 2016). A comparison of miRNA profiles between susceptible and resistant plants can help to dissect the mechanism underlying clubroot resistance. In our previous study, we introduced a dominant clubroot disease resistance locus *(CRb*) into the *B. napus* restorer line Bing 409 and obtained a pair of near-isogenic lines (NILs): a clubroot-resistant line (409R) and a clubroot-susceptible line (409S) (Li et al., 2021). In this study, sRNA-seq and degradome-seq were performed on the roots of 409R and 409S with or without *P. brassicae* infection to identify the critical miRNAs and corresponding target transcripts for clubroot resistance, aiming to establish a model for the miRNA-mediated regulatory network associated with the resistance of *B. napus* to *P. brassicae*.

## Materials and Methods

### Plant Materials

In our previous study, we have introduced the CRb locus from CR Shinki (a Chinese cabbage material) to Bing409 (a Pol. CMS restorer line of *B. napus*) and obtained a pair of NILs with contrast phenotype of clubroot disease resistance through marker-assisted foreground selection and background selection (Li et al., 2021). The resulting lines, namely, the clubroot-resistant line carrying *CRb* locus (designated as 409R) and clubroot-susceptible line (designated as 409S), were sown and grown under a 16-h photoperiod at 25°C in an artificial growth chamber. The single sequence repeat genotyping of 409R revealed that 97% of the recurrent parent genome was recovered.

### *P. brassicae* Inoculation

Rapeseed roots were inoculated with the *P. brassicae* strain collected from Zhijiang (Hubei, China, 30°43'00.00 N, “111°77'00.00” E). The *P. brassicae* strain we used in this study was collected and characterized as pathotypes 4 and Pb1 according to the Williams and sinitic clubroot differential classification systems, respectively (Williams, 1966; Pang et al., 2020). The homogenate of roots with galls or clubs was mixed with dried culture soil at a mass ratio of 1: 20 and sealed at 25°C for more than 48 h. Subsequently, 20 g of the prepared *P. brassica*-containing soil was put into the culture soil in each hole of the cavity tray, which was then filled with tap water (about 40 ml for each hole) and then sown with one to two seeds.

The inoculated roots (Int409R, Int409S) were collected 20 days post-inoculation (dpi), and un-inoculated roots (Mock409R, Mock409S) were collected as control samples. Tissues were immediately frozen in liquid nitrogen and stored at −80°C. The samples were prepared with three biological replicates.

### RNA Extraction and Library Construction for sRNA-seq and Degradome-seq

Total RNA was isolated using the TRIzol (Invitrogen, Carlsbad, CA, United States) reagent according to the instructions of the manufacturer. To ensure the quality of RNA for library construction, an Agilent 2100 Bioanalyzer system was used for RNA quality control. Small RNA (sRNA) was separated from total RNA by NaCl-PEG8000 precipitation, as previously described (Lu et al., 2007). sRNAs in the size range of 18–30 nt were gel-purified and ligated to adapters. The sRNA library was generated by reverse transcription and sequenced using an Illumina HiSeq 2000 platform at Shanghai Personal Biotechnology Co., Ltd in China.

Degradome libraries were generated as previously described (Ma et al., 2010; Zhai et al., 2014). Samples from three biological replicates were pooled for degradome library construction. In brief, mRNA fragments with poly (A) sequences were annealed and captured with poly (T) magnetic beads; 5′ RNA adapters were ligated to RNAs containing 5′ monophosphates. The ligated products were then purified and reverse-transcribed to cDNA using biotinylated random primers. The cDNA was amplified by PCR to construct the degradome libraries. Sequencing was also performed using the Illumina HiSeq 2000 platform at LC-Bio Technologies Co., Ltd (Hangzhou, China).

### Quality Control and Identification of miRNAs

For sRNA sequencing data, the raw reads were first filtered by removal of low-quality reads to obtain clean reads (sRNAs). The clean reads of each sample were screened within a certain range of length, from 18 to 30, and then mapped to the *B. napus* genome (https://www.ncbi.nlm.nih.gov/genome/?term=brassica+napus) using Bowtie to obtain read counts and genomic location information. The sRNAs were also aligned to the GenBank (ftp://ftp.ncbi.nlm.nih.gov/genbank/) and Rfam 11.0 (http://rfam.janelia.org/) databases for functional annotation. All sequences annotated as repeat, intron, exon, ribonucleic acid (rRNA), transfer RNA (tRNA), small cytoplasmic RNA (scRNA), small nuclear RNA (snRNA), or small nucleolar RNA (snoRNA) were removed in subsequent miRNA analyses. Unannotated sRNAs were then used to predict the secondary structure using miReap (http://sourceforge.net/projects/mireap/) combined with genome mapping information. sRNAs with classic miRNA secondary structure were then aligned to miRBase 21.0 (http://www.mirbase.org/ftp.shtml) to identify known miRNAs using miReap. In addition, sRNAs containing classic miRNA secondary structure but not included on miRBase were classified as “novel.” The clean reads for each miRNA were normalized using the following formula: normalized expression (TPM) = mapped read count/total reads ^*^ 1,000,000. Fold changes between the samples were calculated using log_2_ (TPM of sample 1/TPM of sample 2).

### Identification of miRNA Targets

For degradome sequencing data, the raw reads were filtered to remove reads with adapters. Clean reads were aligned to the GenBank and Rfam 11.0 databases to obtain the annotation information for rRNA, tRNA, scRNA, snRNA, and snoRNA. sRNAs not associated with these annotated reads were further mapped to the *B. napus* genome (v2.0, https://www.ncbi.nlm.nih.gov/genome/?term=brassica+napus) to obtain the cDNA sense and antisense reads using Bowtie. The reads mapped to cDNA or mRNA sequences were then used to predict the sites of cleavage. Two software programs were used to predict the sites of cleavage of the targets: psRNATarget (http://plantgrn.noble.org/psRNATarget/) was used to predict miRNA targets; and CleaveLand3 (http://axtell-lab-psu.weebly.com/cleaveland.html) was used to summarize the information of cleaved sites, define categories (containing 0–4 categories), and plot T-plot figures.

### Quantitative RT-PCR and Validation of miRNA Expression

The quantification of miRNAs was performed using miRcute Plus miRNA First-Strand cDNA Synthesis Kit (TIANGEN, China) according to the instructions of the manufacturer. Briefly, 2 μg of total RNA was mixed with an RT RNA reaction buffer and an RT Enzyme mix in a total volume of 20 μl. The reaction system was incubated at 42°C for 60 min and stopped at 95°C for 3 min. quantitative reverse transcription (qRT)-PCR was carried out using the miRcute Plus miRNA qPCR Detection Kit (TIANGEN Biotech Co Ltd, Beijing, China), with miRNA-specific forward primers (Supplementary Table 9) and a universal reverse primer. Briefly, 7.5 μl 2 × miRcute Plus miRNA Premix, 0.3 μl miRNA-specific forward primers, 0.3 μl universal reverse primer, and 6.9 μl 20 × diluted cDNA template were mixed in a total of 15 μl reaction volume. The Bio-Rad CFX96 Realtime System (Bio-Rad, Hercules, CA, United States) was used with the following PCR cycling parameters: 95°C for 15 min; 45 cycles of 94°C for 20 s followed by 60°C for 34 s. Reactions were performed in triplicates, and U6 rRNA was used as the internal reference. The relative expression of miRNAs was calculated according to a previous study (Livak and Schmittgen, 2001). The student's *t*-test was performed for the significance test.

## Results

### Differences in miRNA Pool Between Resistant and Susceptible *B. napus* Upon *P. brassicae* Infection

**Figure 1:**
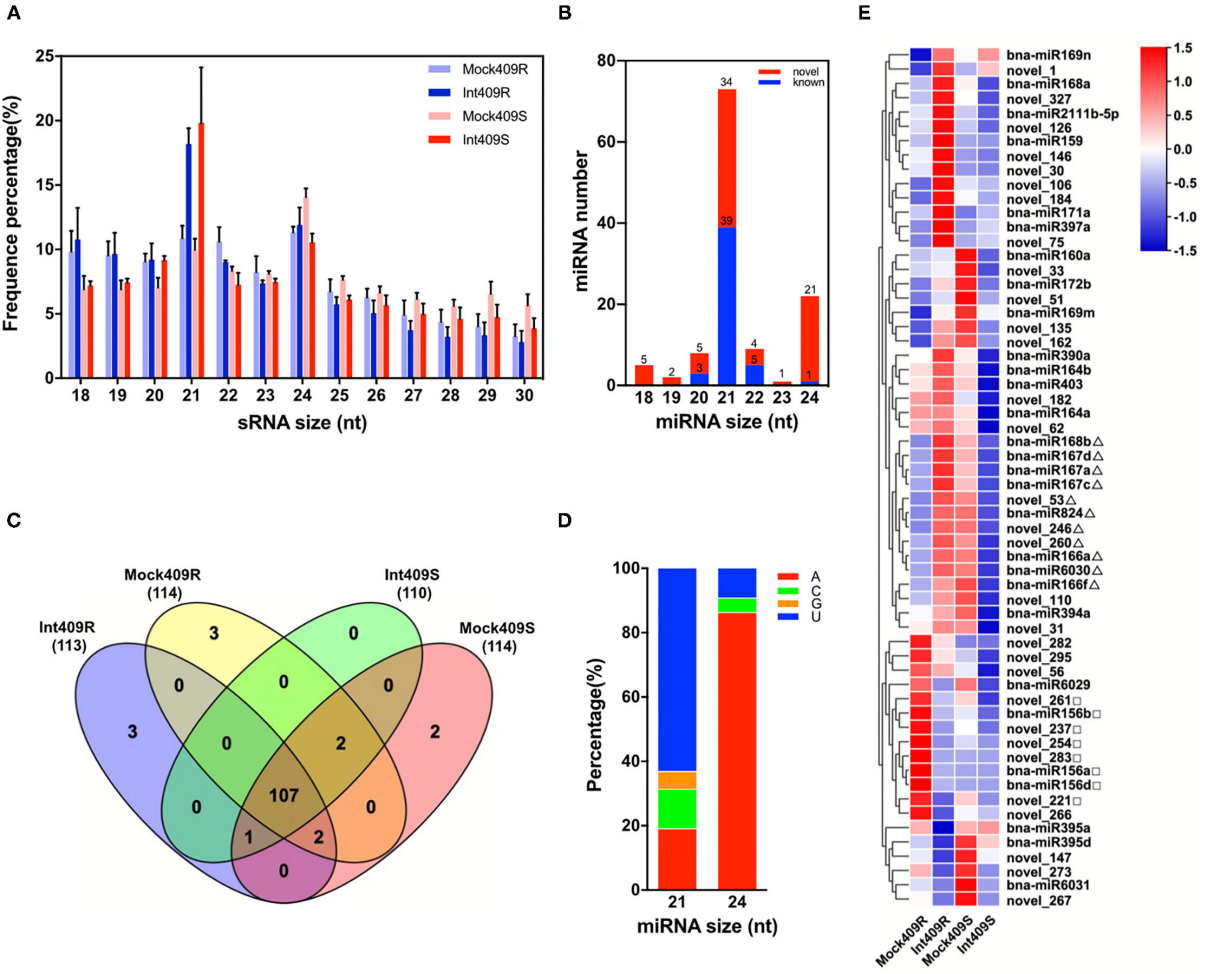
Identification and characterization of micro ribonucleic acids (miRNAs) in resistant and susceptible *Brassica napus* upon *Plasmodiophora brassicae* infection. Distribution of **(A)** Small RNA (sRNA) length in 12 sRNA libraries and **(B)** length distribution of miRNAs, **(C)** Venn diagram of the number of miRNAs in 409R and 409S with or without *P. brassicae* infection, **(D)** preference of the first nucleotide for 21 or 24-nt miRNAs, and **(E)** heat map of 60 highly expressed miRNAs. Δ and □ mark some miRNAs with different expression patterns between 409R and 409S in response to *P. brassicae* infection.

To identify the miRNAs involved in the response of B. napus to *P. brassicae* infection in the resistant line 409R and the susceptible line 409S, 12 sRNA libraries generated from the *P. brassicae* inoculated roots at 20 dpi (Int409R and Int409S) and mock roots (Mock409R and Mock409S) were sequenced with the Illumina Solexa high-throughput sequencing technology. We obtained ca. 1G raw data per sample by SE50 mode (single end, read length 50 nt), with an average of 20.95 million reads (ranging from 17.5 to 40.5 million) for each library (Table 1). About 76.4% of the reads were mapped to the *B. napus* genome, resulting in an average of 16 million clean reads and million repeat reads (Table 1). The reads matched with rRNA, tRNA, snRNA, and snoRNA accounted for 34.25% of the sequences (Table 1). Besides the t/r/sn/snoRNAs, 2.03 million sRNA reads on average were identified from each library (Table 1). Reads corresponding to 18–30 nt sRNAs were selected for further analysis, with the majority of sRNAs exhibiting lengths of 21 and 24 nt (Figure 1A). 409R had relatively more 18-nt and 19-nt sRNAs than 409S; similarly, the inoculated samples (Int409R and Int409S) had more 21-nt sRNAs than the control samples (Mock409R or Mock409S), indicating that *P. brassicae* infection could induce some changes in the sRNA pool of *B. napus* (Figure 1A).

**Table 1:** Statistics for small ribonucleic acid (sRNA) sequencing data.

**Sample**	**Clean reads**	**Mapped reads**	**Unique reads**	**rRNA**	**snRNA**	**snoRNA**	**sRNA**	**Known miRNA**	**Novel miRNA**
Int409R1	16,859,089	12,241,995	2,758,364	1,486,968	19,291	172,035	1,653,701	82,770	21,027
Int409R2	19,437,857	13,583,672	4,032,739	1,211,671	21,381	111,524	2,383,874	113,822	25,952
Int409R3	21,045,591	14,970,123	3,579,575	1,445,208	20,273	125,781	1,935,638	97,756	21,985
Int409S1	19,151,421	12,602,830	2,799,605	1,863,049	7,194	157,600	1,350,982	23,185	5,227
Int409S2	19,510,896	13,667,504	3,262,226	1,374,644	14,476	107,191	1,917,801	81,051	19,851
Int409S3	20,746,687	11,014,507	2,798,106	1,095,158	9,442	72,987	1,391,531	48,203	10,476
Mock409R1	40,544,489	38,503,590	4,241,862	6,659,234	25,051	104,159	3,113,301	42,488	11,810
Mock409R2	19,357,053	12,378,257	3,447,958	1,540,456	14,423	113,443	1,823,459	43,758	15,757
Mock409R3	20,253,468	17,466,642	3,452,723	2,277,865	22,780	143,708	2,313,145	91,820	25,996
Mock409S1	17,482,155	13,689,066	3,305,874	2,014,105	12,946	103,905	1,904,936	47,190	10,243
Mock409S2	17,722,621	15,665,194	3,068,502	1,307,612	30,046	245,728	2,172,936	84,126	24,971
Mock409S3	19,267,299	16,255,438	3,708,198	1,260,635	27,666	115,800	2,383,375	91,333	30,239
Average	20,948,219	16,003,152	3,371,311	1,961,384	18,747	131,155	2,028,723	70,625	18,628

Based on a filtering pipeline designed to distinguish plant miRNAs (Zhai et al., 2011), a total of 120 miRNA precursors were identified, namely, 72 novel miRNAs and 48 known miRNAs (Figures 1B,C, Supplementary Table 1). The lengths of mature miRNAs ranged from 18 to 24 nt, and 21-nt miRNAs (39 known and 34 novel) and 24-nt miRNAs (1 known and 21 novel) were the two most abundant types (Figure 1B). An analysis of nucleotide preference revealed that “U” was preferred by the 21-nt miRNAs, while “A” was preferred by the majority of 24-nt miRNAs (Figure 1D). According to the miRbase database, the 44 known miRNAs belonged to 25 conserved miRNA families across diverse plant species (Supplementary Table 1a). Seventeen novel miRNAs were new members of 11 known miRNA families. For example, novel_147, novel_172, novel_202, novel_207, and novel_222 were new members of the MIR169_2 family (Supplementary Table 1b). A total of 55 novel miRNAs could not be associated with any known miRNA families, and were, thus, defined as “new miRNA candidates” (Supplementary Table 1c). Moreover, five miRNAs (bna-miR1140, bna-miR6032, bna-miR161, bna-miR860, and bna-miR824), which belonged to five miRNA families (MIR1140, MIR6032, MIR161, MIR860, and MIR824), were specifically present in *Brassica* (Table 2, Supplementary Table 2).

**Table 2:** Five micro ribonucleic acids (miRNAs) belonging to miRNA families specific for *Brassica*.

**Mature**	**Precursor**	**miRNA**	**Mature sequence**	**Length**	**Brassica species**
**ID**	**ID**	**Family**		**(nt)**	
bna-miR1140	bna-MIR1140	MIR1140	ACAGCCUAAACCAAUCGGAGC	21	*Brassica napus*
					*Brassica rapa*
bna-miR6032	bna-MIR6032	MIR6032	UGGAGCAUCAACAGAUCUCGG	21	*Brassica napus*
					*Brassica rapa*
bna-miR161	bna-MIR161	MIR161	UCAAUGCACUGAAAGUGACUA	21	*Brassica napus*
					*Brassica rapa*
					*Arabidopsis thaliana*
					*Arabidopsis lyrata*
bna-miR860	bna-MIR860	MIR860	UCAAUACAUUGGACUACAUAU	21	*Brassica napus*
					*Brassica rapa*
					*Arabidopsis thaliana*
					*Arabidopsis lyrata*
bna-miR824	bna-MIR824	MIR824	UAGACCAUUUGUGAGAAGGGA	21	*Brassica napus*
					*Brassica oleracea*
					*Brassica rapa*
					*Arabidopsis thaliana*
					*Arabidopsis lyrata*

There were considerable differences in the expression levels of miRNAs detected in this study (Figure 1E), including highly expressed miRNAs (22.47%) with read numbers of more than 200 across all samples and lowly expressed miRNAs (26.59%) with read numbers below five, while the majority of miRNAs showed read numbers between 5 and 200 (Supplementary Figure 1). Based on the normalized expression of miRNAs, 60 highly expressed miRNAs were selected to construct the heat map for comparing the changes in the miRNAs upon *P. brassicae* infection (Figure 1E). The data revealed that the expression profiles of miRNAs differed greatly between 409R and 409S upon pathogen infection (Figure 1E). For example, bna-miR168b, bna-miR167, bna-miR166, bna-miR824, bna-miR6030, novel_53, novel_260, and novel_246 were upregulated in 409R upon infection, while an opposite trend was observed for 409S (Figure 1E). On the contrary, bna-miR156, novel_261, novel_237, novel_254, novel_221, and novel_283 were downregulated in 409R after infection, while their expression exhibited no change in 409S after *P. brassicae* infection (Figure 1E). Furthermore, 18 miRNAs were significantly differentially expressed in 409R upon *P. brassicae* infection, including nine upregulated (miR168b, miR169m, miR169n, novel98, novel246, novel_75, novel_180, novel_106, and novel_162) and nine downregulated (miR395d, miR6029, novel_221, novel_147, novel_237, novel_295, novel_254, novel_261, and novel_266) (Table 3). In 409S, only one miRNA (novel_1) was found to be upregulated upon *P. brassicae* infection (Table 3).

**Table 3:** Differentially expressed miRNAs in response to *P. brassicae* infection (*p* < 0.05, |log_2_FC| > 1).

**Index**	**miRNA ID**	**Length**	**Int409R vs. Mock409R**		**Int409S vs. Mock409S**	
		**(nt)**	**log_**2**_FC**	***P_***adj***_***	**log_**2**_FC**	***P_***adj***_***
1	novel_1^*^	18			1.36	5.36E-03
2	novel_98	20	2.29	4.17E-03		
3	novel_221	20	−2.14	6.08E-03		
4	novel_246	20	1.04	3.66E-02		
5	bna-miR168b	21	1.31	4.32E-02		
6	bna-miR169m^*^	21	1.41	1.07E-02		
7	bna-miR169n	21	2.37	1.53E-02		
8	Bna-miR395d^*^	21	−1.59	8.95E-03		
9	bna-miR6029	21	−1.62	1.63E-02		
10	novel_75	21	1.04	1.32E-02		
11	novel_147^*^	21	−1.97	3.25E-04		
12	novel_180	21	2.08	1.64E-03		
13	novel_237	21	−3.08	3.78E-03		
14	novel_295	21	−1.49	2.57E-02		
15	novel_106	21	1.45	9.83E-03		
16	novel_162	21	2.34	3.13E-04		
17	novel_254	24	−1.34	1.37E-02		
18	novel_261	24	−1.39	1.77E-02		
19	novel_266	24	−2.73	3.98E-04		

* *miRNAs in bold were experimentally verified by quantitative reverse transcription (qRT)-polymerase chain reaction (PCR)*.

These results suggested that *P. brassicae* infection could cause global changes in the miRNA pool of *B. napus* and that there are substantial differences between the resistant (409R) and susceptible (409S) lines. The differential expression of miRNAs may lead to subsequent changes in target transcripts, which may explain the phenotype differences in plant immune response to *P. brassicae* infection.

### Quantitative RT-PCR Validation of miRNA Expression

**Figure 2:**
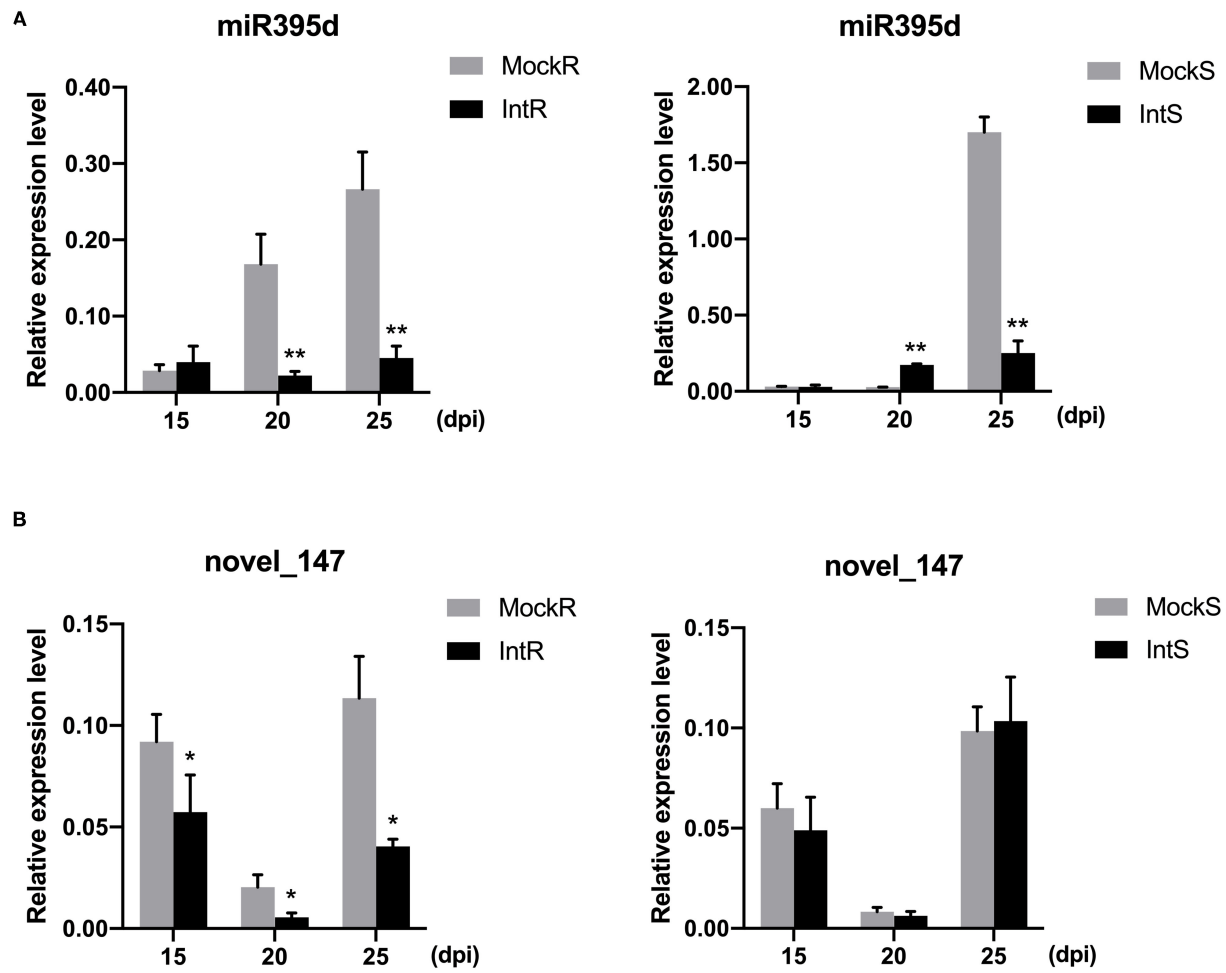
Validation of the relative expression level of **(A)** miRNA395d and **(B)** novel_147 in 409R and 409S in response to *P. brassicae* infection. Y-axis, relative expression level; X-axis, days post-inoculation (dpi). Data were obtained from at least three biological replicates. Bars represent SD (STDEV). *p ≤ 0.05, **p ≤ 0.01 by Student's *t*-test. MockR, 409R without inoculation; IntR, 409R with inoculation; MockS, 409S, without inoculation; IntS, 409S with inoculation.

To validate these results, we examined the expression dynamics of miRNAs at different time points (15, 20, and 25 days) post *P. brassicae* infection by qRT-PCR. The expression levels of four miRNAs were analyzed, and the results are presented in Figure 2. Phase- and species-dependent changes were observed for specific miRNAs. For example, although bna-miR395d was downregulated in both 409R and 409S at 25 dpi, the tendency was different at 20 dpi between 409R and 409S (Figure 2A). In both 409R and 409S, the relative abundance of novel_147 decreased from 15 to 20 dpi and then increased from 20 to 25 dpi, but at each time point upon infection, novel_147 abundance decreased significantly in 409R, while the changes in 409S were not significant (Figure 2B), which was also consistent with the sRNA-seq data (Table 3). Overall, these results indicated dynamic changes in miRNA expression upon *P. brassicae* infection.

### Identification of the Downstream Targets of miRNAs by Degradome Sequencing

Target identification is important for understanding the regulatory function of miRNAs. We constructed four degradome libraries using RNAs derived from Int409R, Int409S, Mock409R, and Mock409S roots to identify the target transcripts of critical miRNAs involved in the progression of clubroot disease. Sequencing of these libraries generated a total of 28 million raw reads and 9 million unique reads on average; 99.52% of the unique reads could be matched to the *B. napus* genome (Supplementary Table 3). As a result, a total of 1,513 miRNA target pairs involving 83 miRNAs (47 known and 36 novel) and 938 target transcripts were identified (Table 4, Supplementary Table 4). The number of target transcripts for a particular miRNA ranged from 1 to 81 (Supplementary Table 5). Interestingly, in some cases, target transcripts could be identified for a subset of miRNAs in 409R but not in 409S and vice versa (Supplementary Table 5). About 63.43% (595/938) of target transcripts showed a one-to-one association with miRNAs, while the rest had two to six matching miRNAs (Supplementary Figure 2). The miRNAs associated with the same transcript usually belonged to the same family (Supplementary Table 6).

**Table 4:** Numbers of target transcripts and miRNA-target pairs identified through sequencing.

**Library**	**miRNAs**	**Transcripts**	**miRNA-Target pairs**	**Category 0**	**Category 1**	**Category 2**	**Category 3**	**Category 4**
Int409R	76	693	1,141	532	45	350	31	183
Mock409R	73	608	1,019	438	56	295	19	211
Int409S	74	613	1,054	474	31	292	25	232
Mock409S	73	619	1,012	466	22	304	24	196
All	83	938	1,513	/	/	/	/	/

**Figure 3:**
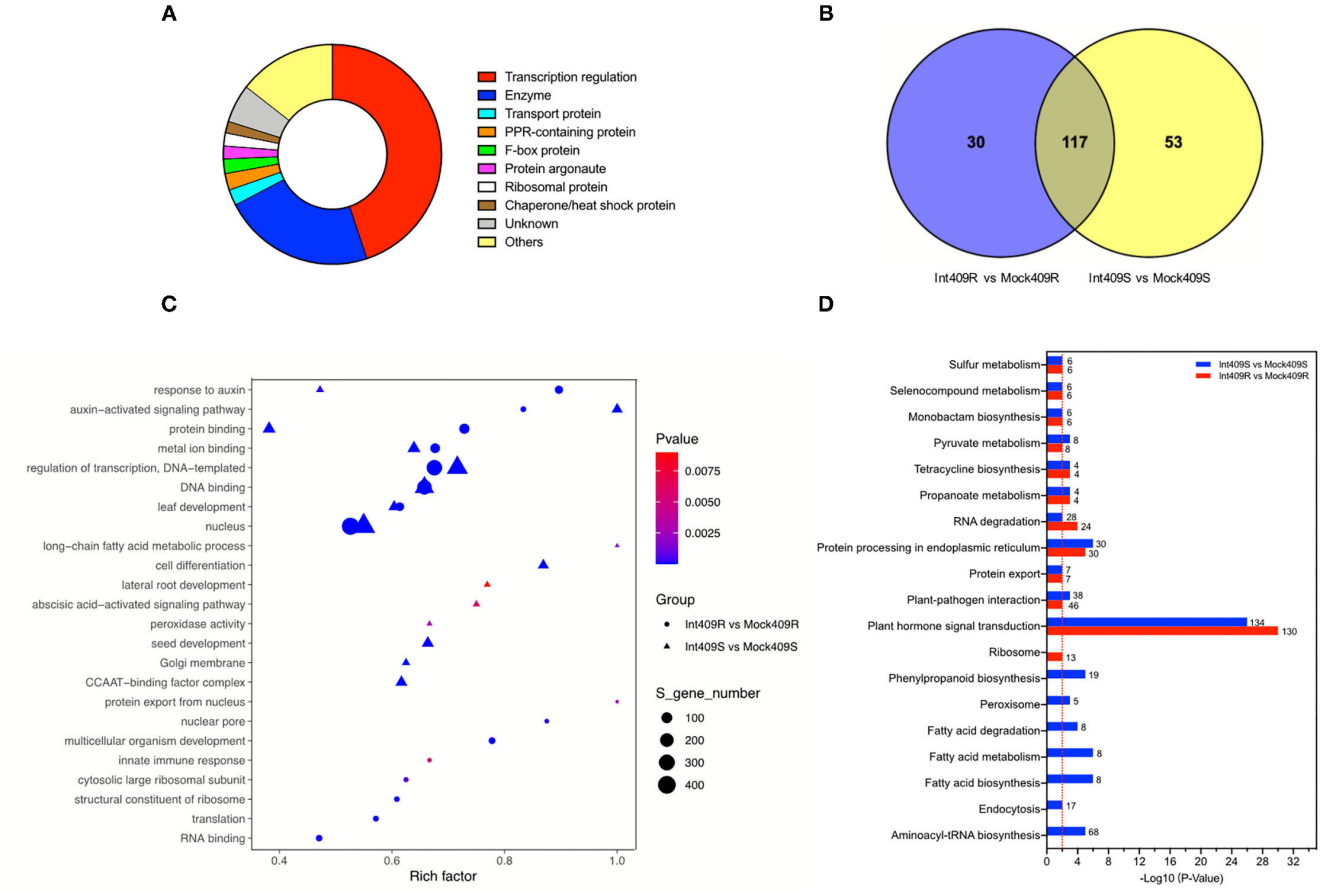
Function annotation and enrichment analysis of target transcripts. **(A)** Functional classification of all detected targets, **(B)** Venn diagram of the number of Gene Ontology (GO) terms, **(C)** GO enrichment, and **(D)** Kyoto Encyclopedia of Genes and Genomes (KEGG) enrichment analyses in comparison between susceptible material (409S) and resistant material (409R) after *P. brassicae* inoculation. Numbers represent the number of target transcripts significantly enriched in the corresponding pathway. The red line means *p* = 0.01.

According to gene function annotation, about 45% (421/938) of the target transcripts were related to transcription regulation (Figure 3A), including transcription factors such as auxin response factors (ARFs), growth-regulating factors (GRFs), ethylene-responsive transcription factors (AP2, TOE, and RAP), myeloblastosis (MYBs), basic helix-loop-helix, Teosinte Branched1-Cycloidea-Pcf, nuclear transcription factor Y subunit As (NFYAs), squamosa promoter-binding-like proteins (SPLs), scarecrow-like proteins (SCLs), and NAC domain-containing proteins (NACs) (Supplementary Table 6). In addition to transcription factors, a variety of enzymes (22.5%; 211/938) and proteins (12.5%; 117/938) involved in several biological processes were also detected by the degradome sequencing (Figure 3A, Supplementary Table 6). These enzymes or proteins participate in diverse cellular processes, namely, signal transduction, lipid transport and metabolism, inorganic ion transport and metabolism, RNA processing, and modification (Figure 3A, Supplementary Table 6).

Gene Ontology (GO) and Kyoto Encyclopedia of Genes and Genomes (KEGG) enrichment analyses were performed to clarify the functions of the targets (Figures 3B–D). In total, 147 and 170 GO terms were significantly enriched in 409R and 409S, respectively, among which 30 and 53 GO terms were differentially enriched upon infection (Figure 3B). The GO enrichment analysis indicated that most of the enriched targets participate in diverse biological processes. The GO terms shared by 409R and 409S included DNA binding, metal ion binding, protein binding, and abscisic acid (ABA)- and auxin-activated signaling pathways. The GO terms specifically enriched in 409S included long-chain fatty acid metabolic process, cell differentiation, lateral root development, peroxidase activity, and ABA signaling pathways (Figure 3C), while different sets of GO terms were found for 409R, such as protein export from the nucleus, nuclear pore, and innate immune response (Figure 3C). These differences between 409R and 409S were also revealed by the KEGG enrichment analysis (Figure 3D). The most significantly enriched KEGG term was “plant hormone signal transduction,” which included 130 and 134 targets in 409R and 409S, respectively (Figure 3D). Seven pathways were uniquely identified for 409S after *P. brassicae* inoculation, namely, “phenylpropanoid biosynthesis, peroxisome, aminoacyl-tRNA biosynthesis, endocytosis, fatty acid biosynthesis, pyruvate and sulfur metabolism, and RNA degradation,” while “ribosome” was the only specific pathway enriched for 409R (Figure 3D, Table 5).

**Table 5:** Micro ribonucleic acids and targets involved in differential enrichment pathways between resistant and susceptible materials.

**Pathway**	**miRNA**	**Target**	**Annotation**
Fatty acid biosynthesis	novel_51	CAC3	*Brassica napus* acetyl-coenzyme A carboxylase carboxyl transferase subunit alpha, chloroplastic
	novel_149	LACS6	*Brassica napus* long chain acyl-CoA synthetase 6, peroxisomal
Fatty acid metabolism	novel_51	CAC3	*Brassica napus* acetyl-coenzyme A carboxylase carboxyl transferase subunit alpha, chloroplastic
	novel_149	LACS6	*Brassica napus* long chain acyl-CoA synthetase 6, peroxisomal
Fatty acid degradation	novel_149	LACS6	*Brassica napus* long chain acyl-CoA synthetase 6, peroxisomal
	novel_283	PP2C38	PREDICTED: *Brassica napus* probable protein phosphatase 2C 38
Peroxisome	novel_149	LACS6	*Brassica napus* long chain acyl-CoA synthetase 6, peroxisomal-like
Endocytosis	novel_283	DRP2A	*Brassica napus* dynamin-2A
	novel_283	DRP2B	*Brassica napus* dynamin-2B
	novel_56	HSP70-6	*Brassica napus* heat shock 70 kDa protein 6, chloroplastic
	novel_51	RABG3B	*Brassica napus* ras-related protein RABG3b
	novel_163	RABH1B	*Brassica napus* ras-related protein RABH1b
	novel_135	TIR-NB-LRR	*Brassica napus* putative disease resistance protein At4g11170
	novel_135	SNC1	*Brassica napus* protein SUPPRESSOR OF npr1-1, CONSTITUTIVE 1-like
	novel_221	EPSIN2	*Brassica napus* clathrin interactor EPSIN 2
Phenylpropanoid biosynthesis	novel_280	C4H	*Brassica napus* trans-cinnamate 4-monooxygenase-like (LOC106348398), mRNA
	novel_320	BGLU15	*Brassica napus* beta-glucosidase 15-like
	novel_51	BGLU44	*Brassica napus* beta-glucosidase 44-like
	novel_54	CAD5	*Brassica napus* cinnamyl alcohol dehydrogenase 5
	novel_30	PER45	*Brassica napus* peroxidase 45
	novel_53	PER69	*Brassica napus* peroxidase 69-like
	novel_217	PER7	*Brassica napus* peroxidase P7-like
Aminoacyl-tRNA biosynthesis	bna-miR169	NFYAs	*Brassica napus* nuclear transcription factor Y subunit A-1/2/3/5/6/9/10
	bna-miR172	AP2	*Brassica napus* floral homeotic protein APETALA 2
Ribosome	novel_1	RPL13B	*Brassica napus* 60S ribosomal protein L13-1-like
	novel_1	RPL13C	*Brassica napus* 60S ribosomal protein L13-2
	novel_259	RPL23A	*Brassica napus* 60S ribosomal protein L23a-2
	novel_280	RPS18A	*Brassica napus* 40S ribosomal protein S18

### miRNA-Target Pairs Involved in Resistance to *P. brassicae*

**Figure 4:**
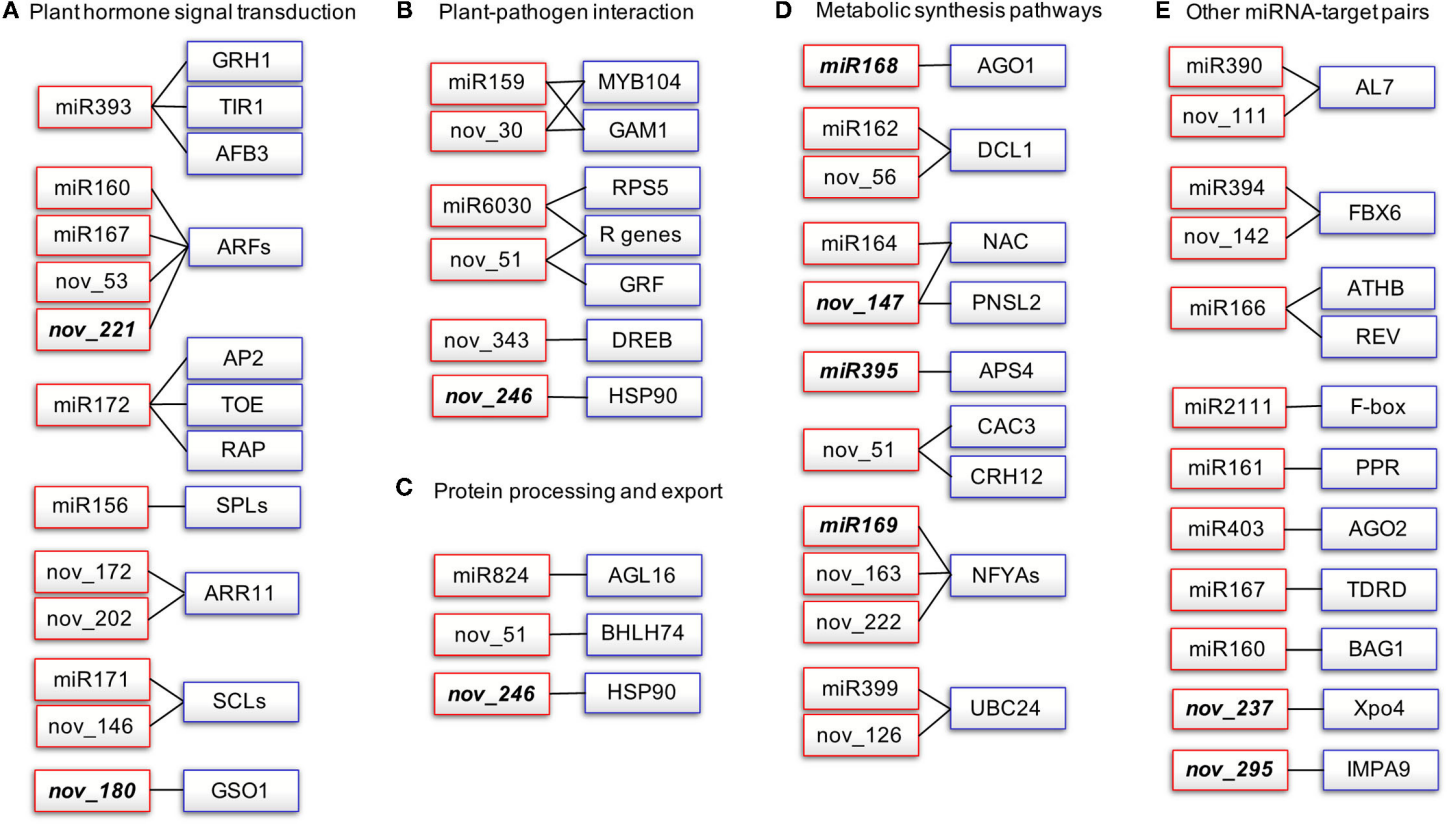
Network of degradome validated miRNA-target pairs associated with *P. brassicae* response in *B. napus*. Red rectangles represent the miRNAs and blue rectangles represent the target transcripts validated by degradome sequencing. Italics indicate miRNAs that are significantly differentially expressed after *P. brassicae* infection. AFB, auxin signaling F-box; AGO1, argonaute1; AP2, apetala2; APS, ATP sulfurylase; ARF, auxin response factor; MYB, myeloblastosis; NFYA, nuclear transcription factor Y subunit alpha; AGL, agamous-like MADS-box protein; AL7, PHD finger protein ALFIN-LIKE 7-like; BAG1. BAG family molecular chaperone regulator 1-like; CAC3, acetyl-coenzyme A carboxylase carboxyl transferase subunit alpha; DCL1, endoribonuclease Dicer homolog 1; DREB, dehydration-responsive element-binding protein 2B-like; FBX6, F-box only protein 6; GRF, growth-regulating factor; HSP90, heat shock protein 90-2-like; PNSL2, photosynthetic NDH subunit of lumenal location 2, chloroplastic-like; PPR, pentatricopeptide repeat-containing protein; SCL, scarecrow-like protein; SPL, quamosa promoter-binding-like protein; TIR1, transport inhibitor response 1. **(A)** Plant hormone signal transduction, **(B)** Plant-Pathogen interaction, **(C)** Protein processing and export, **(D)** Metabolic synthesis pathways, **(E)** Other miRNA-target pairs.

To gain deeper insights into the defense mechanisms of *B. napus* against *P. brassicae* infection, we selected the miRNA-target pairs with degradome category values below 2 and we constructed a miRNA-target network that included five subclusters based on functional category annotation (Figure 4, Supplementary Table 7).

A total of 16 kinds of miRNA-target pairs (cluster I) were associated with plant hormone signal transduction (ko04075, *P*-value: 1.48E-30; Figure 4A, Supplementary Table 7), namely, miR393-TIR1/AFB3/GRH1 and miR160/miR167/novel_53/novel_221-ARFs for auxin signaling, miR172-AP2/TOEs/RAP2-7 for ethylene response, miR156-SPLs for jasmonic acid (JA) signaling, and novel_172/novel_202-ARR11 for type-A response regulators in response to cytokinin (CK). miR171 and novel_146 both target SCLs required for quiescent center cell specification and maintenance in root meristem zone, and asymmetric cell division for radial pattern formation. Novel_180 targets GSO1 (protein brassinosteroid in sensitive 1-like), which is involved in the regulation of root development and root morphogenesis (Figure 4A, Supplementary Table 7). Cluster II comprised 10 pairs of miRNA-targets associated with plant-pathogen interaction (ko04626), namely, miR159 and novel_30 targeting both GAM1 and MYB104, miR6030, and novel_51 targeting both RPS5 and R genes, novel_246 targeting HSP90-2, and novel_343 targeting DREB2B (Figure 4B, Supplementary Table 7). Cluster III included three miRNA-target pairs for protein processing and export (ko04141, *P*-value: 9.07E-06; Figure 4C). Cluster IV consisted of miRNA-target pairs involved in metabolic synthesis pathways (Figure 4D): miR168 targets AGO1; miR162 and novel_56 target DCL1; both AGO1 and DCL1 are involved in RNA-mediated post-transcriptional gene silencing (PTGS); miR164 and novel_147 both target NACs involved in RNA degradation; novel_147 targets PNSL2 involved in photosynthesis; miR395 targets APS for sulfate-deficiency response, seleno-compound metabolism, sulfur metabolism, and monobactam biosynthesis; novel_51 targets CAC3 for fatty acid biosynthesis and metabolism, propanoate metabolism, pyruvate metabolism, and tetracycline biosynthesis; miR169, novel_163, and novel_222 target NFYAs involved in aminoacyl-tRNA biosynthesis; and miR399 and novel_126 target UBC24 associated with ubiquitin-mediated proteolysis (Figure 4D). miRNA-target pairs that did not fit into any of clusters I–IV are listed in cluster V (Figure 4E, Supplementary Table 7).

### Expression Profiles of miRNA-Target Pairs Responsive to *P. brassicae*

Combined with the transcriptome data, the expression profiles of both miRNAs and their targets responsive to *P. brassicae* infection were integrated to infer the regulatory role of miRNAs during *P. brassicae* infection. We obtained a total of 27 differentially expressed targets of eight miRNAs responsive to *P. brassicae* upon infection in 409R and 409S, with a cutoff value of *p* < 0.05 and |log_2_FC| of > 1 (Supplementary Table 8). To be more specific, there were six antagonistic miRNA-target pairs in 409R upon infection, such as miR395d-NM_001315829.1 (APS4), miR395d-XM_013888737.2 (uncharacterized), miR395d-XM_013820969.2 (uncharacterized), novel_147-XM_013818148.2 (NAC076) and novel_147-XM_013820748.2/XM_013857207.2 (PNSL2) (Table 6). Some targets cleaved by miRNAs were validated by degradome sequencing (Supplementary Figure 3).

**Table 6:** Differentially expressed miRNA-target pairs in response to *P. brassicae* (*p* < 0.05, |log_2_FC| > 1).

**miRNA**	**Log_**2**_FC**	**Target transcript**	**Gene ID**	**Log_**2**_FC**	**Symbol**	**Annotation**
**A. Int409R vs Mock409R**						
bna-miR169m	1.41	XM_013829031.2	LOC106388859	1.52	NFYA2	PREDICTED: *Brassica napus* nuclear transcription factor Y subunit A-2-like
		XM_013880479.2	LOC106439112	1.65	NFYA2	PREDICTED: *Brassica napus* nuclear transcription factor Y subunit A-2-like
bna-miR169n	2.37	XM_013880479.2	LOC106439112	1.65	NFYA2	PREDICTED: *Brassica napus* nuclear transcription factor Y subunit A-2-like
		XM_013843872.2	LOC106403032	1.16	NFYA3	PREDICTED: *Brassica napus* nuclear transcription factor Y subunit A-3-like
		XM_013843696.2	LOC106402883	1.36	NFYA3	PREDICTED: *Brassica napus* nuclear transcription factor Y subunit A-3
		XM_013846894.2	LOC106406270	1.22	NFYA3	PREDICTED: *Brassica napus* nuclear transcription factor Y subunit A-3 -like
		XM_013817829.2	LOC106377560	1.17	NFYA6	PREDICTED: *Brassica napus* nuclear transcription factor Y subunit A-6
		XM_013794135.2	LOC106354243	1.48	NFYA3	PREDICTED: *Brassica napus* nuclear transcription factor Y subunit A-3-like
bna-miR395d	−1.59	XM_013893754.2	LOC106451780	−1.30	APS1	PREDICTED: *Brassica napus* ATP sulfurylase 1, chloroplastic-like
		XM_013797400.2	LOC106357712	−1.15	APS1	PREDICTED: *Brassica napus* ATP sulfurylase 1, chloroplastic-like
		**NM_001315829.1**	LOC106410303	0.98	APS4	*Brassica napus* ATP sulfurylase 4, chloroplastic
		**XM_013888737.2**	LOC106446918	1.45	/	PREDICTED: *Brassica napus* uncharacterized
		**XM_013820969.2**	BNAC04G40010D	1.79	/	PREDICTED: *Brassica napus* uncharacterized BNAC04G40010D
novel_147	−1.97	**XM_013818148.2**	LOC106377933	2.84	NAC076	PREDICTED: *Brassica napus* NAC domain-containing protein 76-like
		**XM_013820748.2**	LOC106380909	3.15	PNSL2	PREDICTED: *Brassica napus* photosynthetic NDH subunit of lumenal location 2, chloroplastic-like
		**XM_013857207.2**	LOC106416345	2.75	PNSL2	PREDICTED: *Brassica napus* photosynthetic NDH subunit of lumenal location 2, chloroplastic-like
**B. Int409S vs Mock409S**						
novel_1	1.36	**XM_013876125.2**	LOC106435262	−1.42	/	PREDICTED: *Brassica napus* uncharacterized protein At2g33490-like
		**XM_013887915.2**	LOC106446212	−1.94	/	PREDICTED: *Brassica napus* uncharacterized protein At2g33490-like
		**XM_022705170.1**	LOC106402738	−1.22	/	PREDICTED: *Brassica napus* uncharacterized protein At2g33490-like

*Bold letters indicate that target transcripts are negatively regulated by corresponding miRNAs*.

## Discussion

### miRNA Participates in the Response of *B. napus* to *P. brassicae* Infection

Numerous studies have demonstrated that miRNAs are involved in plant-pathogen interactions. In this study, we systematically studied the regulatory roles of miRNAs and their targets in response to *P. brassicae* infection using the resistant line 409R and the susceptible line 409S of rapeseed, which possessed the same genetic background while exhibiting a contrasting phenotype of clubroot resistance. The aim of the study is to identify key candidate miRNAs and corresponding target transcripts involved in clubroot resistance of *B. napus*. The findings may build up a better understanding of the regulatory network underlying the immune response of *B. napus* to clubroot pathogen infection.

We noticed some differences in the abundance of particular miRNAs by sRNAseq compared with qRT-PCR (data not shown). Although these results were not presented, but might be encountered for similar studies using qRT-PCR to validate sRNAseq results. Difference RT strategies have been used for RNAseq and qRT of miRNAs, allowing discrimination between pri-, pre- and mature miRNAs (Verma et al., 2014; Wei et al., 2016). The cellular level of pri- or pre- precursors are very low and therefore difficult to quantify, in the current study we focus on the expression level of mature miRNAs and their regulation on target transcripts relevant for clubroot disease progression.

Although these results were not presented, they still might be anticipated for similar studies using both sRNAseq and qRT-PCR to quantify the abundance of miRNAs. These differences could be generated because of the different RT strategies used for RNAseq and qRT, and, indeed, might also reveal a difference in the cellular level of pri-, pre-, and mature miRNAs. As we know, miRNAs made from a primary transcript goes through processing to yield stem-loop structured pre-miRNA. Mature miRNA is generated by the Ago complex that results in 21- to 24-nt single-stranded sRNAs. A change in the mature miRNA level could be generated through pri- or pre- precursors; however, they are present in low abundance, easily degraded in defined cellular compartments and, therefore, difficult to quantify.

The sRNA-seq data revealed that 21-nt and 24-nt sRNAs accounted for the highest proportion of the total sRNAs. Interestingly, after *P. brassicae* inoculation, the relative abundance of 21-nt sRNAs increased in both 409R and 409S (Figure 1A). The miRNAs identified in this study included members of known miRNA families of rapeseed, as well as 17 novel members of conserved miRNA families and 55 completely new miRNAs (Supplementary Table 1). The identification of these new miRNAs expanded the current knowledge of the miRNA pool in *B. napus*. Interestingly, there were 18 differentially expressed miRNAs (DE miRNAs) in 409R after *P. brassicae* infection, while there was only one DE miRNA in 409S (Table 3). Many of the DE miRNAs are novel candidates that have not been reported before (Supplementary Table 1). The fact that the resistant line (409R) had more DE miRNAs than its isogenic counterpart (409S) upon infection highlights a possible regulatory role of R genes in clubroot disease resistance.

### Fatty Acid Metabolism Might Participate in the Interaction Between Susceptible *B. napus* and *P. brassicae*

The GO and KEGG enrichment analyses identified pathways related to fatty acid metabolism in 409S (Figure 3C, Table 5). Two miRNA-target pairs associated with lipid biosynthesis pathways, novel_51-CAC3 and novel_149-LACS6, were identified only in 409S upon infection (Figure 3C, Table 5). It has been reported that resting spores of *P. brassicae* can accumulate lipid droplets as an energy source for future sporulation and that many genes related to the fatty acid metabolism of *P. brassicae* have been identified (Bi et al., 2016). It has also been shown that fatty acids, as carbon source nutrients, play an important role in plant-powdery mildew interaction (Jiang et al., 2017). We speculate that fatty acids also serve as a potential determinant of the interaction between *P. brassicae* and *B. napus*. *P. brassicae* might take advantage of plant fatty acid biosynthesis for successful and systematic invasion. More studies are needed to unravel the function of fatty acid biosynthesis in clubroot disease progression.

### Regulatory Network of miRNA-Targets on Diverse Cellular Pathways in Resistant *B. napus* Responding to *P. brassicae* Infection

**Figure 5:**
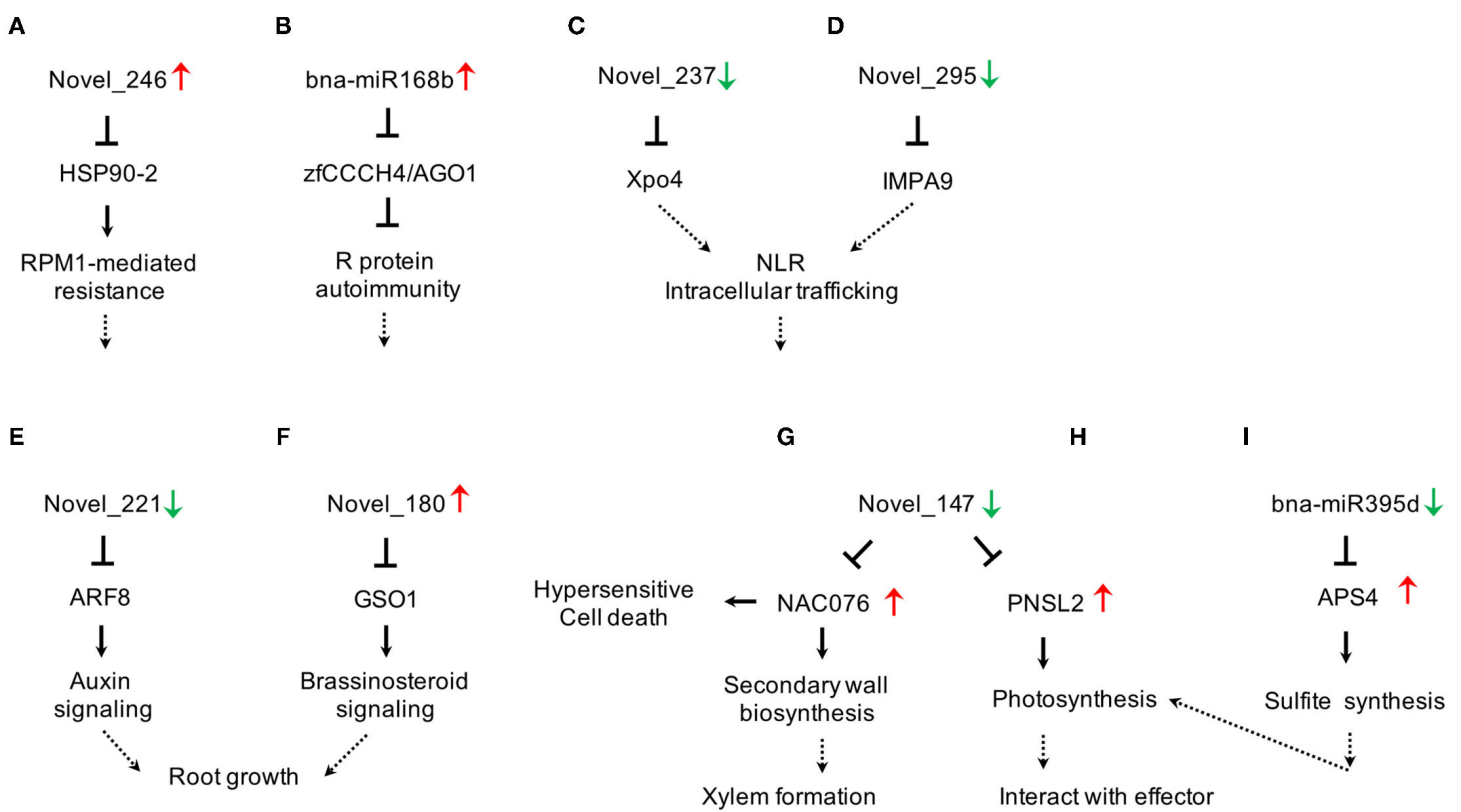
Diverse cellular pathways regulated by miRNA-target modules in clubroot-resistant *B. napus* in response to *P. brassicae*. **(A,B)** Novel_246-HSP90-2 and bna-miRA168b-zfCCCH4/AGO1 for miRNA biogenesis; **(C,D)** Novel_237-Xpo4 and novel_295-IMPA9 for NLR signalling; **(E,F)** Novel_221-ARF8 and Novel_180-GSO1 for root growth; **(G,H)** Novel_147-NAC076/PNSL2 for secondary wall biosynthesis and photosynthesis; **(I)** bna-miR395d-APS4 for sulphite synthesis. Up- or Down- regulation of miRNAs or target transcripts were indicated by red or green arrows, respectively.

We found that the abundance of miRNAs changed more dramatically in 409R than in 409S upon infection (Table 3). Combining transcriptome and degradome data, we constructed a miRNA-target regulatory network in clubroot-resistant *B. napus* in response to *P. brassicae* infection (Figure 5). miRNA biogenesis is known to be important for PTI response (Agorio and Vera, 2007; Navarro et al., 2008), but relevant miRNAs have not been identified in *B. napus*. In the novel_246-HSP90-2 pair (Figure 5A), novel_246 was upregulated in 409R (Table 3), and HSP90-2 encoded a molecular chaperone that regulates RPM1/RPP4-mediated defense response (Bao et al., 2014; Huang et al., 2014). Similarly, bna-miR168b was upregulated in 409R (Figure 5B), which targets the AGO1 and Zinc finger transcription factor CCCH4. In *Malus hupehensis*, miR168 targets AGO1 and contributes to resistance against *Botryosphaeria dothidea* infection (Yu et al., 2017). Therefore, some of these identified DE miRNAs may regulate the target key transcripts/proteins through miRNA interference silencing complex (miRISC) to mediate host response to clubroot disease.

nucleotide binding and leucine rich repeat (NLR) proteins are nucleic acid-binding proteins involved in pathogen-induced signaling (Jones and Dangl, 2006). Two NLR-type R genes for *P. brassicae* have been identified (Hatakeyama et al., 2013, 2017). Many studies have shown that NLRs are localized to both the cytoplasm and nucleus and that their nuclear accumulation is necessary for pathogen resistance (Shen et al., 2007; Bai et al., 2012; Inoue et al., 2013). Importins and exportins, which act as transport receptors, play important roles in the nuclear pore complex (NPC)-directed partitioning of nucleocytoplasmic NLRs (Garcia and Parker, 2009; Meier and Somers, 2011). In this study, two novel miRNAs (novel_237 and novel_295) that target exportins4 (Xpo4) and importin subunit alpha-9-like (IMPA9) were found to be downregulated in 409R upon infection (Table 3, Figure 4E). It can be inferred that the changes in these miRNAs and further in importins and/or exportins can affect the nucleocytoplasmic partitioning of NLRs. NLRs function together with other cellular metabolic or signaling pathways to contribute to the disease resistance of 409R after *P. brassicae* inoculation (Figures 5C,D).

The root is the source organ for *P. brassicae* infection, and its morphology and development, especially those of root hair and root cortex, are critical for clubroot disease progression (Kageyama and Asano, 2009). ARF8 is known as an auxin response factor that inhibits root elongation and promotes lateral root initiation (Wang et al., 2015). We found that novel_221, which regulates ARF8, was downregulated in 409R after *P. brassicae* infection (Table 3, Figure 5E). Similarly, the novel_180-GSO1 pair is associated with root growth (Figure 5F). GSO1 works in coordination with GSO2 to regulate root growth through cell division and specification (Racolta et al., 2014; Nakayama et al., 2017). Our data suggest that the abundance of novel_180 increased in 409R after *P. brassicae* infection (Table 3). Therefore, novel_221-ARF8 and novel_180-GSO1 might regulate root growth and suppress gall formation in 409R *via* hormone signaling (Figures 5E,F).

Plant secondary cell wall thickening is a powerful way to prevent the systematic spreading of pathogens after being attacked. NAC-containing protein is a master transcription activator for xylem formation and SCW thickening (Zhou et al., 2014). Another study has reported that *Arabidopsis* miR164a and its target NAC4 play important roles in regulating hypersensitive (HR) cell death in response to avirulent bacterial pathogens (Lee et al., 2017). In our research, NAC076 expression was also under the control of novel_147 and miR164 (Figure 4D). We found that novel_147 was downregulated in 409R after *P. brassicae* infection, and NAC076 transcript level was also upregulated accordingly (Table 6). The activation of NAC076 could lead to the induction of SCW genes and subsequent cell wall thickening or HR cell death, which will then block the invasion of *P. brassicae* and confer disease resistance (Figure 5G).

Another target of novel_147 was PNSL2, which acts as a chloroplast NAD(P)H dehydrogenase (NDH) complex (Marjaana et al., 2010; Shinya et al., 2010; Figure 5H). Some studies have suggested interplay between photosynthesis and plant defense (Xu et al., 2011; Rodríguez-Herva et al., 2012). In *Arabidopsis*, the PSII subunit PsbP interacts with the coat protein of the *Alfalfa mosaic* virus to inhibit viral replication (Balasubramaniam et al., 2014). In another case, *P. syringae* effectors, HopI1 and HopN1, can remodel host chloroplasts by interacting with PsbQ of PSII to suppress immunity response (Jelenska et al., 2007). In this study, with the downregulation of novel_147 in 409R upon infection, the transcripts of PNSL2 were upregulated (Table 6). We hypothesize that miRNAs competitively target and modulate photosynthesis-related genes in chloroplasts, thereby indirectly inhibiting proteins interacting with *P. brassicae* effectors and then mediating resistance to *P. brassicae* in 409R. Lastly, the miR395d-APS4 pair may also mediate clubroot disease progression *via* photosynthesis (Figure 5I). Inorganic sulfate from the soil is absorbed by root hairs and then transported to leaves, where it is activated into adenosine 5'-phosphosulfate by APSs (ATP sulfurylases) in chloroplasts (Liang et al., 2010; Jagadeeswaran et al., 2014). We found that after *P. brassicae* infection, 409R showed a decrease in the abundance of bna-miR395d and an increase in the transcript level of APS4 (Table 6). The effect of sulfite synthesis on chloroplast physiology and disease progression in roots remains to be further explored (Figure 5I).

## Data Availability Statement

The data presented in the study are deposited in the BioSample repository (https://www.ncbi.nlm.nih.gov/biosample/), accession number PRJNA742780. Accessions from SAMN19977475-SAMN19977486 are data from sRNAseq; accessions SAMN19977487-SAMN19977490 are data from degradome sequencing.

## Author Contributions

QL analyzed the data, performed the experiments, prepared the figures, and drafted the manuscript. XZ helped analyzed the data. CZ and PC conceived the study and participated in its coordination. PC helped to draft the manuscript. All authors have read and approved the final version of the manuscript.

## Funding

This study was supported by the National Natural Science Foundation of China (Grant No: 31871659) and CARS-12 to CZ.

## Conflict of Interest

The authors declare that the research was conducted in the absence of any commercial or financial relationships that could be construed as a potential conflict of interest.

## Publisher's Note

All claims expressed in this article are solely those of the authors and do not necessarily represent those of their affiliated organizations, or those of the publisher, the editors and the reviewers. Any product that may be evaluated in this article, or claim that may be made by its manufacturer, is not guaranteed or endorsed by the publisher.
