# Supplementary material for: Identification of Micro Ribonucleic Acids and Their Targets in Response to Plasmodiophora brassicae Infection in Brassica napus
Source: Front Plant Sci. 2021 Oct 28;12:734419. doi: 10.3389/fpls.2021.734419 (PMC8585624; doi:10.3389/fpls.2021.734419)
Supplement: Supplementary file 10 [file Data_Sheet_1.DOCX]

Supplementary Material

# Supplementary Data

# Supplementary Figures and Tables

## Supplementary Figures

##
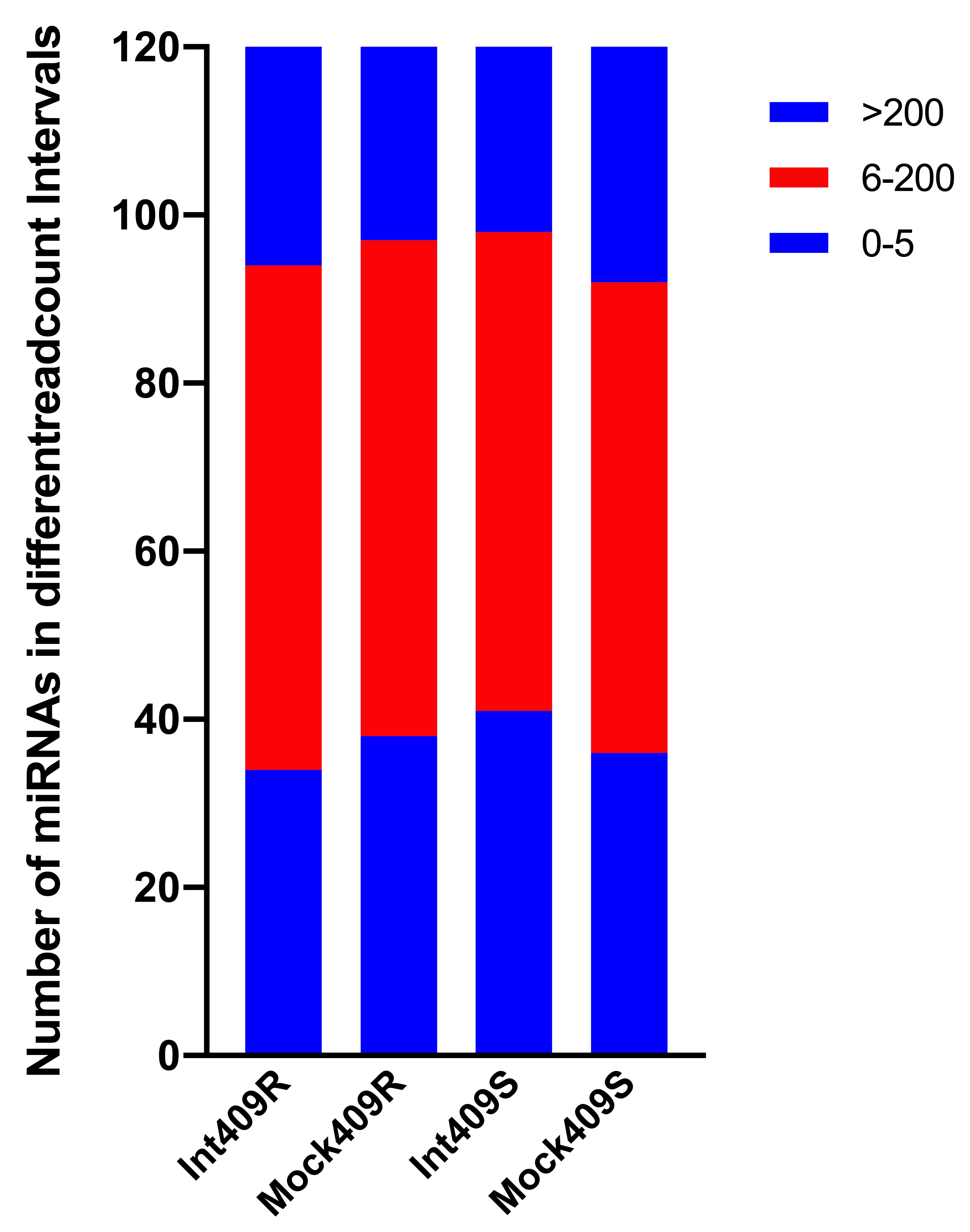


## Supplementary Figure S1 The number of miRNAs in different readcount intervals.

**Supplementary Figure S2** Different types of regulatory relationship between miRNA and target transcripts *in B.napus.*

**Supplementary Figure S3** T-plots and miRNA-mRNA alignments validated by degradome sequencing where miR395d cleaves APS4 (NM_001315829.1) gene (A); novel_147 cleaves NAC76 (XM_013818148.2) gene (B); novel_147 cleaves PNSL2 (XM_013820748.2) gene (C). The red line and arrows represent the cleavage nucleotide positions on the target genes.
